# Proteomic analysis of sex differences in hyperoxic lung injury in neonatal mice

**DOI:** 10.7150/ijms.42073

**Published:** 2020-09-02

**Authors:** Huaiping Cheng, Huifang Wang, Chantong Wu, Yuan Zhang, Tianping Bao, Zhaofang Tian

**Affiliations:** Department of Neonatology, The Affiliated Huaian No.1 People's Hospital of Nanjing Medical University; the Pediatric Diagnosis and Treatment Respiratory Key Laboratory of Huai'an, Huai'an 223300, China.

**Keywords:** bronchopulmonary dysplasia, hyperoxia, tandem mass tags, lung tissues, gender, proteomics

## Abstract

Sex-specific differences in the severity of bronchopulmonary dysplasia (BPD) are due to different susceptibility to hyperoxic lung injury, but the mechanism is unclear. In this study, neonatal male and female mouse pups (C57BL/6J) were exposed to hyperoxia and lung tissues were excised on postnatal day 7 for histological analysis and tandem mass tags proteomic analysis. We found that the lung sections from the male mice following postnatal hyperoxia exposure had increased alveolar simplification, significant aberrant pulmonary vascularization and arrest in angiogenesis compared with females. Comparison of differentially expressed proteins revealed 377 proteins unique to female and 425 unique to male as well as 750 proteins in both male and female. Bioinformatics analysis suggested that several differentially expressed proteins could contribute to the differences in sex-specific susceptibility to hyperoxic lung injury. Our results may help identify sex-specific biomarkers and therapeutic targets of BPD.

## Introduction

Bronchopulmonary dysplasia (BPD) affects about 40% of neonates born before 28 weeks globally 1. BPD is mainly caused by maternal eclampsia and ventilation and is characterized by alveolar and microvascular injury and pulmonary hypertension 2. Despite improved perinatal care, the incidence of BPD is still high 3. Therefore, better understanding of molecular mechanisms of BPD is important for the prevention and treatment of BPD.

Sexual dimorphism has been observed in such respiratory diseases as BPD, asthma, chronic obstructive pulmonary disease, pulmonary hypertension, lung cancer and pulmonary fibrosis 4-9. The male has higher risk of BPD in premature neonates 10. Variations among male and female premature newborns in the progression and outcome of BPD are intimately linked to physiological, hormonal and structural differences 11. Moreover, increased expression of cytochrome p450 may contribute to improved outcomes in female mice 12. A recent study showed that miR-30a was implicated in sexual dimorphism in BPD 13.

Proteomics is a promising approach for clinical and diagnostic applications by mapping proteomes from cells, tissues, and organisms for the discovery of new disease biomarkers 14. Label-free liquid chromatography-mass spectrometry (LC-MS/MS) has been applied to reveal the pathogenesis of BPD 15. Tandem mass tags (TMTs) have been applied in gel-free proteomic approach 16. In this study, we employed TMTs approach to compare lung proteomic profiles of neonatal mice of different sex exposed to hyperoxia.

## Materials and Methods

### Animals

All animal experiments were performed at The Affiliated Huaian No.1 People's Hospital of Nanjing Medical University (Huaian, China) and the protocols were approved by Animal Use and Care Committee of The Affiliated Huaian No.1 People's Hospital of Nanjing Medical University. A total of 32 C57BL/6J neonatal mouse pups were purchased from Animal Center of Nanjing Medical University (Nanjing, China). The sex of mice was determined by physiological observation and PCR analysis on postnatal day 7 (PND7) for Sry gene.

### BPD model

Thirty-two mouse pups (16 males and 16 females) within 6 h of birth were randomly assigned to two groups for the exposure to room air (21% O_2_) and hyperoxia (FiO_2_ ≥ 95%) for 7 days, respectively. Hyperoxia exposure was performed in Plexiglas chambers allowing continuous monitoring and constant oxygen content (95-100% oxygen). The details of the establishment of BPD model were described previously 17. At the end, all mice were anesthetized by sodium pentobarbital, sacrificed and the lungs were excised.

### Histology analysis

The chest cavity was opened and the heart was inflated with phosphate buffered saline until the lungs turned white. The left and right upper lungs were kept at -80°C immediately. The rest of the lungs were fixed at 4°C with 4% paraformaldehyde and embedded in paraffin. The tissue was cut into sections (5 μm) and stained with haematoxylin-eosin. Radial alveolar counts (RAC) were evaluated as described previously 17. Fifteen randomly chosen fields were observed with a 10× objective of a microscope in a blinded fashion.

### Protein extraction, peptide digestion and labeling

Protein extraction and peptide digestion were performed as described previously 18. Briefly, lung tissues were lysed in RIPA lysis buffer (Keygen Biotech), and the proteins in lysates were concentrated and digested with Trypsin Gold (Promega) for 16 h at 37°C. The digested peptides were desalted with C18 cartridge and dried by vacuum. The peptides were then labeled with TMT6/10plex™ Isobaric Label Reagent (Thermofisher) following the manufacturer's instructions. The labeled peptides were desalted using peptide desalting spin columns (ThermoFisher, 89852).

### LC-MS/MS analysis

The labeled peptides were fractionated using C18 column (Waters BEH C18 4.6×250 mm, 5 μm) at 50°C. Mobile phases A was 2% acetonitrile (pH 10.0) and B was 98% acetonitrile (pH 10.0). The solvent gradient was: 3% B, 5 min; 3-8% B, 0.1 min; 8-18% B, 11.9 min; 18-32% B, 11 min; 32-45% B, 7 min; 45-80% B, 3 min; 80% B, 5 min; 80-5%, 0.1 min,5% B, 6.9 min. The samples were dried by vacuum and dissolved in 0.1% (v/v) formic acid. Next the samples were subjected to shotgun proteomics analyses on EASY-nLCTM 1200 UHPLC system (ThermoFisher) coupled to an Orbitrap Q Exactive HF-X mass spectrometer (ThermoFisher) as described previously 18.

### Bioinformatics analyses

Bioinformatics analyses were performed as described previously 19, including hierarchical clustering analysis, gene ontology (GO) analysis (based on http://www.geneontology.org/), KEGG (Kyoto Encyclopedia of Genes and Genomes) pathway analysis (based on http://www.genome.jp/kegg/), enrichment analysis (based on Fisher's Exact Test).

### Enzyme-linked immunosorbent assay (ELISA)

To confirm the differentially expressed proteins identified by LC-MS/MS, we focused on FABP4 (fatty-acid-binding protein 4) and CXCL4 (Chemokine C-X-C motif ligand 4). Lungs were homogenized in PBS (pH 7.4) and centrifuged at 10,000×g to remove insoluble debris. The levels of FABP4 and CXCL4 in lung tissues were detected by ELISA kit (Thermo Fisher Scientific, Shanghai, China) according to the manufacturer's instructions.

### Statistical analysis

Data were displayed as mean ± stand derivation (SD) and analyzed by two-way ANOVA. *p* < 0.05 was considered significant.

## Results

### Increased alveolar simplification in male mice exposed to hyperoxia exposure

**Figure 1:**
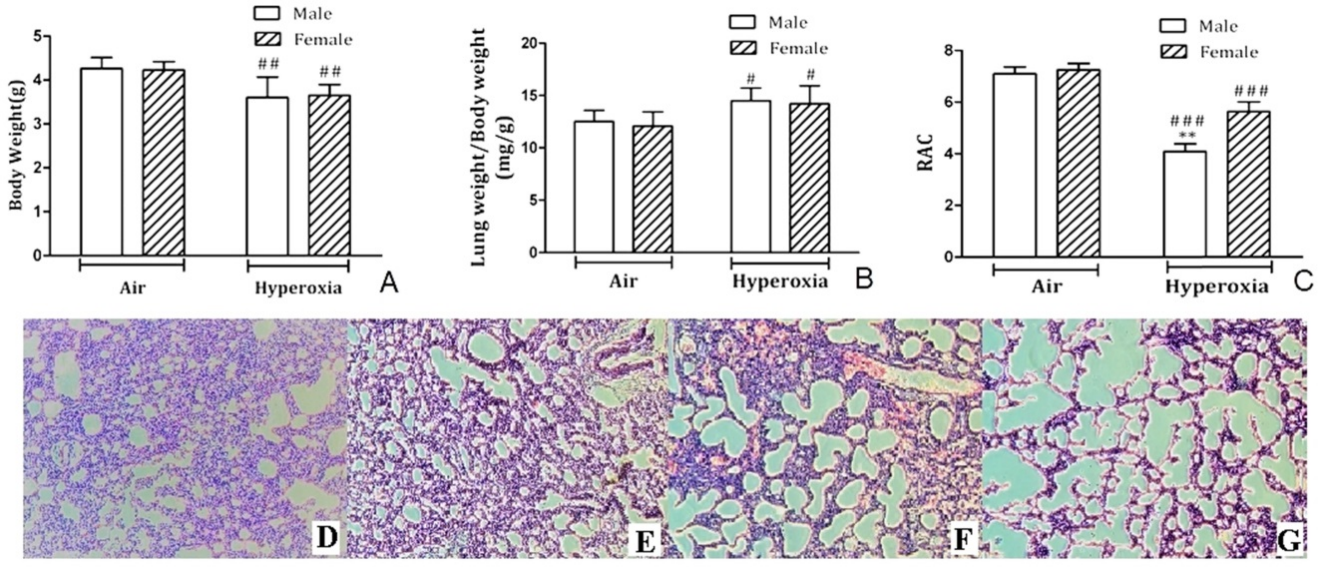
** Histological analysis of lung tissues. A.** Body weights of male and female postnatal day (PND) 7 mice exposed to hyperoxia or room air (n=8/group). ##*P*<0.01. **B.** Lung weight/Body weight ratios (mg/g) in male and female neonatal mice exposed to hyperoxia on PND7 immediately after hyperoxia exposure (n=8/group). #*P*<0.05. **C.** Radial alveolar count in male and female neonatal mice exposed to room air or hyperoxia on PND 7. **P<0.01. ###*P <*0.001. All values are mean ± SD (n=8). D. H&E staining of the lungs in female neonatal mice exposed to air. **E.** H&E staining of the lungs in male neonatal mice exposed to air. **F.** H&E staining of the lungs in female neonatal mice exposed to hyperoxia. **G.** H&E staining of the lungs in male neonatal mice exposed to hyperoxia. (×100 magnification).

No animals in hyperoxia exposed group or control group died during the experiment. Newborn mouse pups were weighed on PND7, and mice showed significant weight loss (*p* < 0.01) after exposure to 95% O_2_ for 7 days compared to room air controls (Fig. 1A). However, body weights were not different for hyperoxia-male and female mice on PND7 (*p* > 0.05). Moreover, lung weight/body weight (LW/BW) ratio increased in both male and female neonatal pups with no difference (Fig. 1B). RAC was measured to assess postnatal alveolarization. On PND7, no significant difference was observed in RAC of male and female pups kept in room air, but RAC was significantly different in male mice and female mice exposed to hyperoxia (Fig. 1C). H&E staining showed typical alveolar simplification, decreased alveolar surface area and mild interstitial thickening after hyperoxia exposure, especially in male neonatal mice (Fig. 1D-G).

### Hyperoxia led to gender differences in protein expression patterns

Compared to room air group, 1,557 proteins displayed significant expression changes (≥1.2 fold, *p* < 0.05) in hyperoxia group. In this cohort, 638 proteins were upregulated and 919 proteins were downregulated. Compared with the room air controls, 4 of the 1,557 identified proteins, including Myosin-4, Heavy polypeptide 7, MCG140437 and Alpha-actinin-3, were upregulated in hyperoxia-exposed males but were downregulated in hyperoxia-exposed females. Notably, fructose-bisphosphate aldolase was downregulated in hyperoxia-exposed males but was upregulated in hyperoxia-exposed females compared to control group.

**Figure 2:**
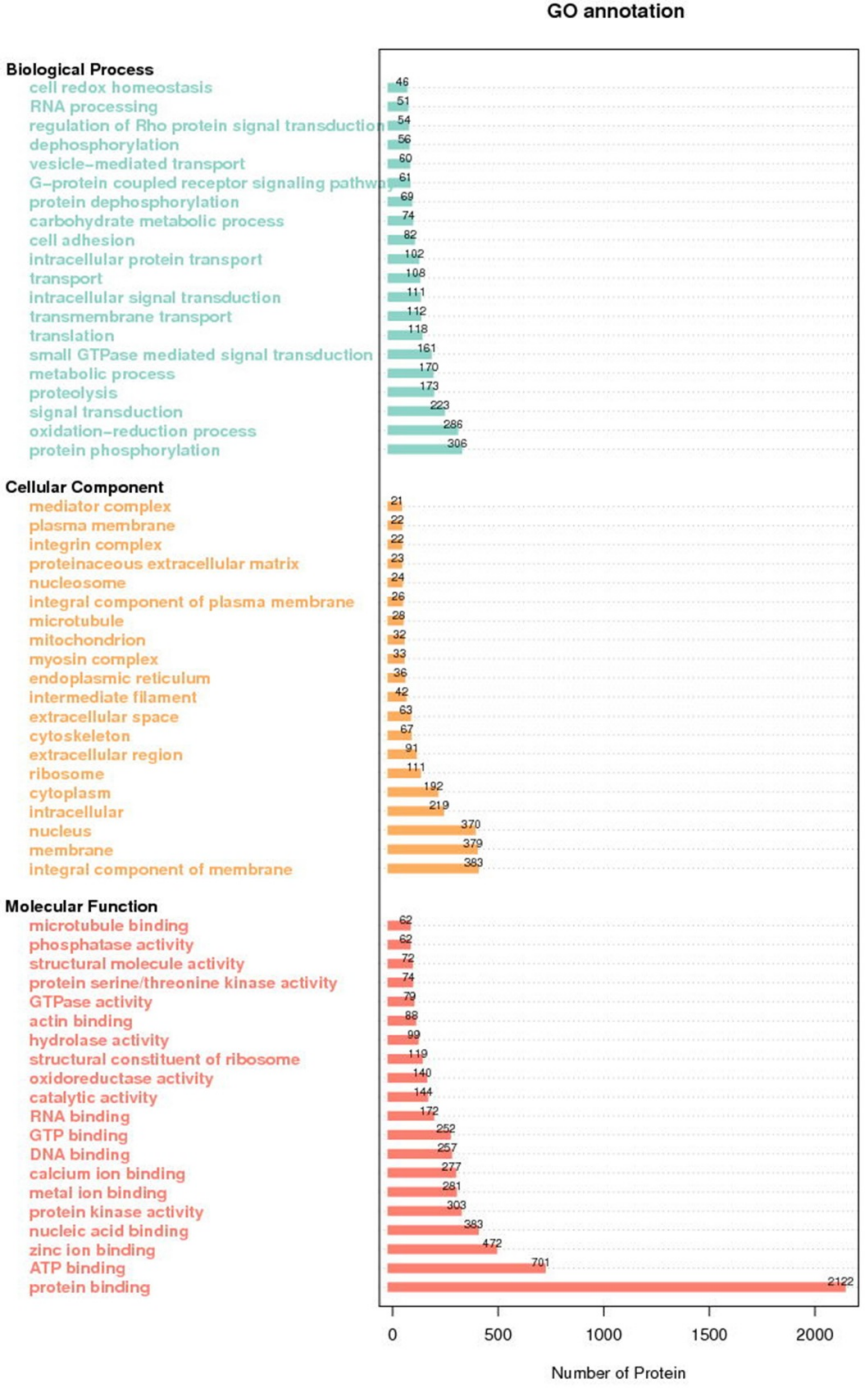
GO annotation of all the differentially expressed proteins.

GO analysis of all identified proteins showed their functional classifications (Fig. 2). In particular, we found oxidation-reduction process, proteolysis and protein phosphorylation in biological process classification, protein binding, ATP binding and zinc binding in molecular function classification, and membrane, nucleus and integral component of membrane in cellular protein classification.

### Differentially expressed proteins between hyperoxia and room air exposed mice in both sexes

**Figure 3:**
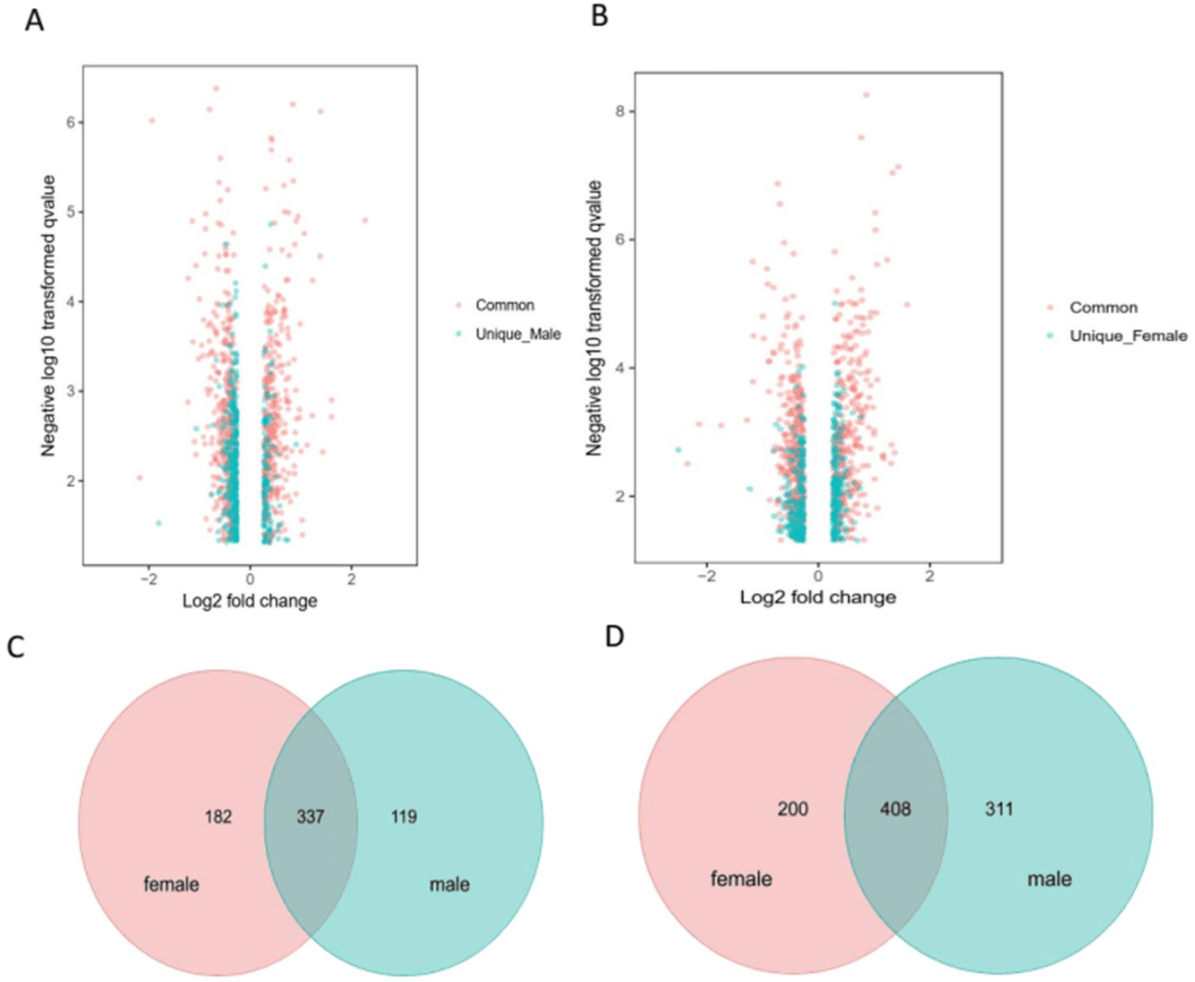
** Distinct pulmonary proteomic patterns in male and female neonatal mice after exposure to hyperoxia.** A-B. Volcano plots of the differentially expressed proteins (DEPs) in male and female neonatal mice in response to hyperoxia exposure compared to room air controls at PND7. The proteins shaded in red are common DEPs between male and female neonatal mice, whereas the proteins represented in blue are DEPs exclusive to male (A) and exclusive to female (B) neonatal mice. Venn diagrams highlighting the upregulated 182 proteins in female mice only, 119 proteins in male mice only, and 337 proteins in both male and female mice (C), downregulated 200 proteins in female mice only, 311 proteins in male mice only, and 408 proteins in both male and female mice) (D) in male and female neonatal mice (PND7) exposed to hyperoxia compared to room air controls.

To limit the intrinsic gender influence, we performed the analysis of differentially expressed proteins by comparing hyperoxia-exposed group of males or females with gender-matched room air-group. Fig. 3A & B showed the volcano plot in male and female mice, respectively. Venn diagrams highlighting the upregulated 182 proteins in female mice only, 119 proteins in male mice only, and 337 proteins in both male and female mice (total 638 upregulated proteins) (Fig. 3C), as well as downregulated 200 proteins in female mice only, 311 proteins in male mice only, and 408 proteins in both male and female mice (total 919 downregulated proteins) (Fig. 3D) exposed to hyperoxia compared to room air controls.

Eight of the 750 proteins present significant differences between the two genders, including Alpha-1-acid glycoprotein 1, MCG1051009, Pentaxin, Alpha-1-antitrypsin 1-5, Gene trap ROSA b-geo 22, Alpha-S1-casein, MCG121569 and Exocyst complex component 3-like protein 4. The level of upregulation of Alpha-1-acid glycoprotein 1, MCG1051009, Pentaxin, Alpha-1-antitrypsin 1-5 and Gene trap ROSA b-geo 22 was significantly higher in hyperoxia-exposed males than in females, whereas the relative expression abundance of Alpha-S1-casein, MCG121569 and Exocyst complex component 3-like protein 4 was significantly lower in hyperoxia-exposed females.

**Figure 4:**
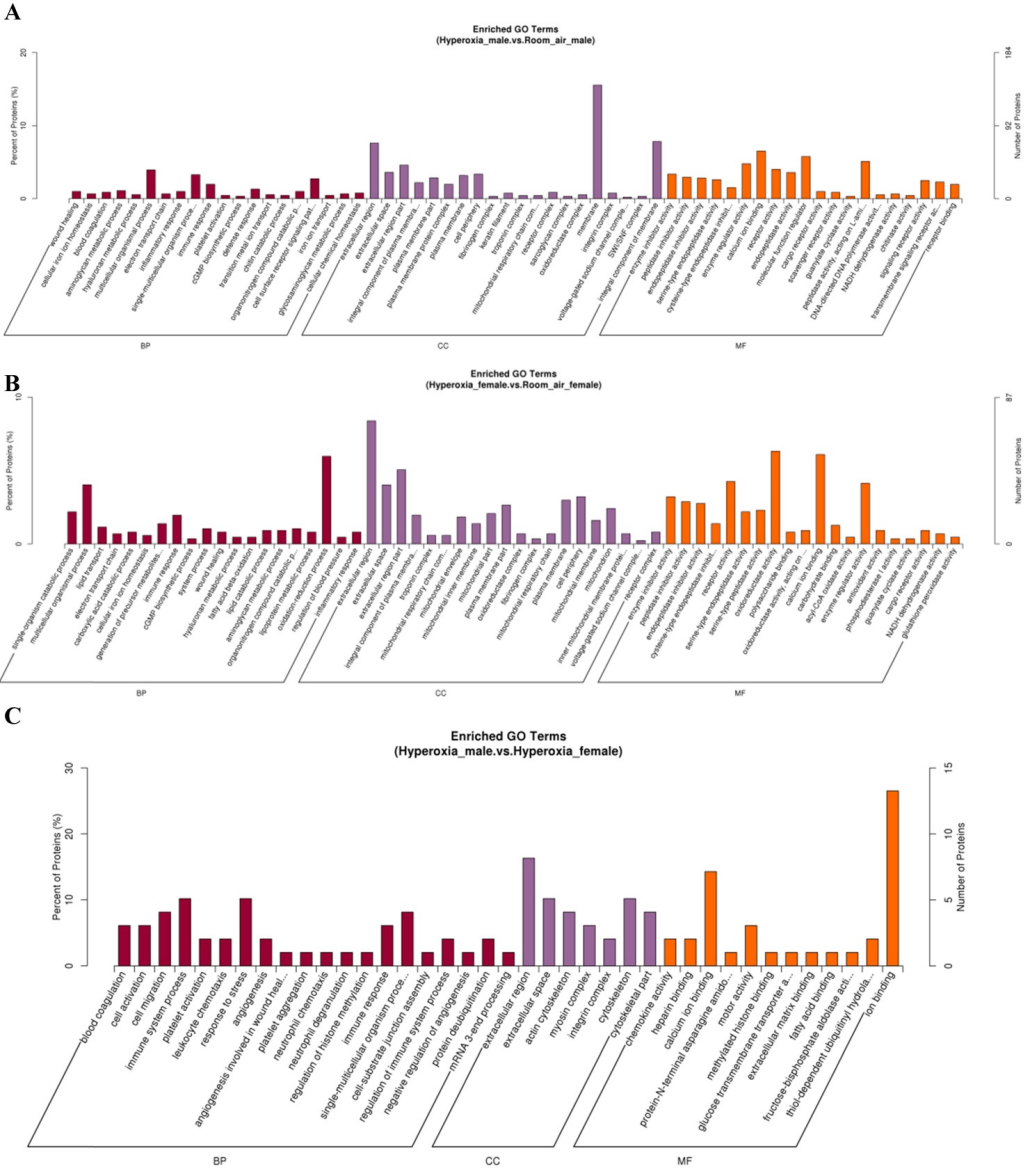
** Gene ontology (GO) analysis of differentially expressed proteins. A.** DEPs only in male mice. **B.** DEPs only in female mice. C. DEPs common in male and female mice. BP: biological process; CC: cellular component; MF: molecular function.

**Figure 5:**
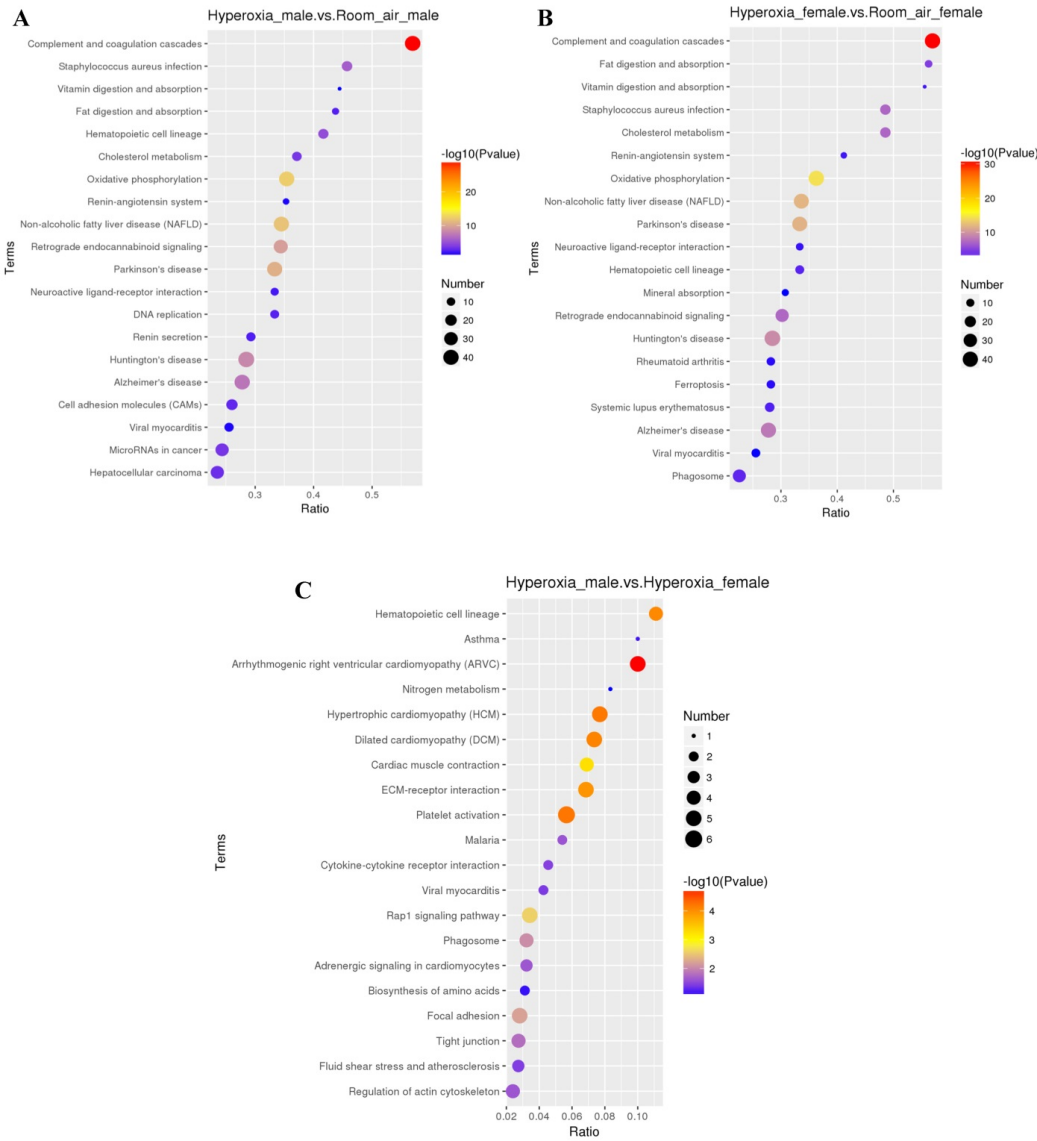
** KEGG analysis of differentially expressed proteins. A.** DEPs only in male mice. **B.** DEPs only in female mice. **C.** DEPs common in male and female mice.

GO analysis showed that the differentially expressed proteins were involved in several biological processes, including response to multicellular organismal process, immune system process and single-multicellular organism process. The most significantly affected molecular functions were calcium ion binding, enzyme regulator activity, and molecular function regulator (Fig. 4A-C). Pathway analysis by KEGG suggested that the top significantly affected pathways were complement and coagulation cascades and oxidative phosphorylation (Fig. 5A-C).

### Differentially expressed proteins between hyperoxia and room air exposed male mice

Total 425 proteins were exclusively identified with significant alteration in abundance (≥1.2 fold, *p*< 0.05) in males in hyperoxia group. 118 proteins were upregulated while 307 proteins were downregulated**.** GO analysis (Fig. 4A) revealed that molecular functions of these proteins mainly include protein binding, DNA binding, protein complex binding and actin filament binding. The mainly affected cellular component were nucleus, plasma membrane part and plasma membrane protein complex. The mainly affected biological processes were cell adhesion, chromatin remodeling and ATP-dependent chromatin remodeling, which was consistent with those of KEGG analysis. Pathway analysis by KEGG suggested that the top 4 significantly affected canonical pathways were P53 signaling pathway, P13k-Akt signaling pathway, EGFR tyrosine kinase inhibitor resistance, and axon guidance (Fig. 5A).

### Differentially expressed proteins between hyperoxia and room air exposed female mice

For females, 377 proteins were exclusively identified to be significantly altered (≥1.2fold, *p* < 0.05) in the hyperoxia-exposed group. Gene ontology analysis (Fig. 4B) revealed that they also involve in several biological processes, the top three of which are response to stress, carbohydrate metabolic process and single-organism catabolic metabolic process. The top 5 most significantly affected molecular functions were oxidoreductase activity, structural constituent of ribosome, antioxidant activity, glutathione peroxidase activity and acyl-CoA oxidase activity. Pathway analysis by KEGG suggested that the top 4 significantly affected canonical pathways were ribosome, fluid shear stress and atherosclerosis, phagosome, and amino sugar and nucleotide sugar metabolism (Fig. 5B).

### Validation of changed CXCL4 and FABP4 levels in the lungs of hyperoxia exposed mice

**Figure 6:**
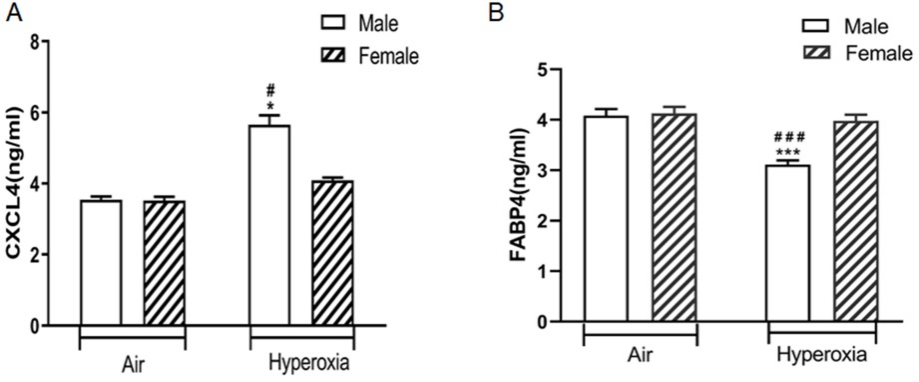
** The levels of CXCL4 and FABP4 in lung homogenates of different groups of mice. A.** CXCL4 level. #*P <*0.05 Significant differences between room air and hyperoxia exposed mice. *P<0.05 Significant differences between male and female mice. **B.** FABP4 level. ###*P <*0.001 Significant differences between room air and hyperoxia exposed mice. ***P<0.001 Significant differences between male and female mice. Data are presented as mean ±SD (n=8).

ELISA analysis showed that CXCL4 was exclusively differentially upregulated in the lungs of hyperoxia exposed neonatal male mice compared to hyperoxia exposed female mice (*p <* 0.05) and air exposed neonatal male mice (*p <* 0.05). There was no significant difference in CXCL4 level in the lungs between hyperoxia exposed and room air exposed female mice (*p >* 0.05) (Fig. 6A). In contrast, FABP4 was exclusively differentially downregulated in the lungs of hyperoxia exposed neonatal male mice compared to hyperoxia exposed female mice (*p <* 0.05) and air exposed neonatal male mice (*p <* 0.05). There was no significant difference in FABP4 level in the lungs between hyperoxia exposed and room air exposed female mice (*p >* 0.05) (Fig. 6B).

## Discussion

To our knowledge, our study is the first report on sex specific proteomic changes in hyperoxic lung injury. Using TMT approach, we screened differentially expressed proteins unique and common to both sexes. Our preliminary data showed baseline differences in lung proteome in male and female newborn mice exposed to room air (data not shown because we found large number of differentially expressed proteins). In this study we focused on the differences in lung proteome in male and female newborn mice exposed to hyperoxia. Among the differential proteins, based on the literatures we selected FABP4 and CXCL4 for further validation of changed protein levels by ELISA.

FABP4 is proposed as an adipokine produced in adipocytes, macrophages and endothelial cell, and is involved in the regulation of macrophages and airway inflammation 20. FABP4 deficient mice were protected from atherosclerosis 21. FABP4 participated in leucocyte infiltration into the airway 22. Moreover, serum levels of FABP4 increased in many diseases including bronchopulmonary dysplasia 23. Furthermore, serum FABP4 levels in female COPD patients increased significantly 24. In our study, FABP4 was downregulated in male mice after hyperoxia exposure, which partially explains why neonatal male mice are more susceptible to hyperoxia-mediated lung injury. Chemokine C-X-C motif ligand 4 (CXCL4) is a pleiotropic chemokine involved in blood coagulation, angiogenesis, tumor and immune system regulation 25,26. In our study, CXCL4 was exclusively upregulated in hyperoxia exposed neonatal male mice. CXCL4 regulates the differentiation of monocytes by inducing a specific macrophage phenotype termed “M4”. M4 macrophages are a subgroup of macrophages that express high levels of proinflammatory factors such as TNF-α and IL-6 27. IL-6 and TNF-α are known to be involved in sex specific hyperoxic lung injury 28.

Pathway analysis showed sex specific differences in biological processes. Similar dysregulation in protein homeostasis and PI3K-Akt signaling pathway have been described in hyperoxic lung injury 29, 30. Our findings are similar to the study performed in autopsy lung samples of preterm neonates on cell-cycle regulation, immune-cell regulation and sonic hedgehog signaling as differentially regulated in babies with BPD 31. Notably, p53 pathway was upregulated in male mice while oxidative phosphorylation was downregulated in females. These data may represent ongoing apoptosis and oxidative stress in male mice, which may contribute to impaired lung development.

We also identified differentially expressed proteins common to both sexes. Calsequestrin-1 (CASQ1) is an acidic protein which binds Ca^2+^ with moderate affinity but high capacity 32,33. We found that CASQ1 was downregulated in both male and female mice. Dainese et al. showed that the incidence of mortality in CASQ1-null mice during exposure to both halothane and heat was significantly greater in males than in females 34. The difference in the susceptibility of CASQ1-null mice could reside in their different abilities to modulate oxidative stress 33. The overproduction of reactive oxygen species could cause damages to lung epithelial cells 35.

In summary, we report pulmonary proteomics in mice exposed to hyperoxia. Our results show sex specific modulation of protein expression and biological processes. Comprehensive analysis of proteomic profiling would provide new insights into sex specific differences in the pathogenesis of hyperoxia induced lung injury.
